# A Novel Ras--Related Signature Improves Prognostic Capacity in Oesophageal Squamous Cell Carcinoma

**DOI:** 10.3389/fgene.2022.822966

**Published:** 2022-02-24

**Authors:** Hao-Shuai Yang, Wei Liu, Shao-Yi Zheng, He-Yuan Cai, Hong-He Luo, Yan-Fen Feng, Yi-Yan Lei

**Affiliations:** ^1^ Department of Thoracic Surgery, The First Affiliated Hospital, Sun Yat-sen University, Guangzhou, China; ^2^ State Key Laboratory of Oncology in South China, Collaborative Innovation Center for Cancer Medicine, Sun Yat-sen University Cancer Center, Guangzhou, China; ^3^ Department of Pathology, Sun Yat-sen University Cancer Center, Guangzhou, China

**Keywords:** oesophageal squamous cell carcinoma, ras, prognosis, bioinformatics analysis, TCGA, GTEx

## Abstract

Oesophageal squamous cell carcinoma (ESCC) remains a clinically challenging disease with high morbidity rates and poor prognosis. ESCC is also the most common pathological type of oesophageal cancer (EC) in China. Ras-related genes are one of the most frequently mutated gene families in cancer and regulate tumour development and progression. Given this, we investigated the Ras-related gene expression profiles and their values in ESCC prognosis, using data from the Genotype-Tissue Expression (GTEx) and The Cancer Genome Atlas (TCGA) databases. We found that we could identify three distinct oesophageal cancer clusters based on their unique expression profile for 11 differentially expressed Ras-related genes with each of these demonstrating some prognostic value when, evaluated using univariate Cox analysis. We then used multivariate Cox analysis to identify relevant independent prognostic indicators and used these to build a new prognostic prediction model for oesophageal cancer patients using these three Ras-related genes. These evaluations produced an area under the curve (AUC) of 0.932. We found that our Ras-related signatures could also act as independent factors in ESCC prognosis and that patients with low Ras scores showed a higher overall expression levels of various immune checkpoint genes, including TNFSF4, TNFRSF8, TNFRSF9, NRP1, CD28, CD70, CD200, CD276, METTL16, METTL14, ZC3H13, YTHDF3, VIRMA, FTO, and RBM15, as well as a higher CSMD3, FLG, DNAH5, MUC4, PLCO, EYS, and ZNF804B mutation rates, and better sensitivity to drugs such as erlotinib, paclitaxel, and gefitinib. In conclusion, we were able to use the unique expression profiles of several Ras-related genes to produce a novel disease signature which might facilitate improved prognosis in ESCC, providing new insight into both diagnosis and treatment in these cancers.

## Introduction

Ras proteins are a class of GTPase that function as molecular switches in various signalling pathways regulating cellular proliferation, differentiation, survival, migration, and cytoskeletal dynamism. It has been shown that Ras-related genes are the most commonly mutated gene family in cancer, and Ras activation resulting from mutations in Ras genes or their regulators promotes the development and progression of a variety of cancers. (Pylayeva-Gupta et al., 2011; Simanshu et al., 2017; Moore et al., 2020). Oesophageal carcinoma (EC) is the seventh most common cancer globally with the sixth worst prognosis. Oesophageal squamous cell carcinoma (ESCC) and Oesophageal adenocarcinoma (EAC) are the two major subtypes of EC and are classified based on their pathology, showing obvious differences in incidence, aetiology and clinical characteristics. (Arnold et al., 2015). ESCC is the most common pathological type of EC in China, accounting for approximately 90% of all cases. Most patients are clinically diagnosed at an advanced stage and present with a very poor prognosis, with a 5-years overall survival (OS) of only 18.8%. (Abnet et al., 2018; Arnold et al., 2015). The effect of surgery alone for advanced ESCC is far from satisfactory, because of its high recurrence rates and poor survival. (Bray et al., 2018). Therefore, multidisciplinary treatment is highly recommended to improve prognosis and several recent studies have shown that the Ras signalling pathway plays an important role in the pathogenesis of EC. To the best knowledge of our, few studies have explored the relationship between Ras and ESCC.

Here, we systematically profiled the genomic information from both ESCC and normal samples using their clinical outcomes from the Genotype-Tissue Expression (GTEx) and Cancer Genome Atlas (TCGA) databases. This study was designed to investigate Ras gene expression profiles and their value in ESCC prognosis. We then used this information construct a novel prognostic prediction model for oesophageal cancer patients based on their Ras-related gene profile, providing a new tool for the diagnosis and treatment of ESCC.

## Methods and Materials

### Oesophageal Cancer Dataset Source and Pre-Processing

Transcriptome gene expression, mutation frequency of transcriptome genes, and clinical data from ESCC patients were downloaded from the GTEx (https://commonfund.nih.gov/gtex) and TCGA databases (https://portal.gdc.cancer.gov/). We then controlled for bias by excluding, patients with missing genetic data in the GTEx database and five patients without sufficient clinical follow-up information from the TCGA database. Finally, we downloaded the original “CEL” files for the microarray data from Affymetrix, and applied a robust multiarray averaging method to produce our data set and then downloaded, the “limma” file and completed a robust multiarray averaging method to produce the dataset needed to complete the necessary background adjustment and quantile normalization of this datest in R version 4.0.2. We then downloaded the standardised matrix files directly for microarray data from other platforms and all data were quantile-normalized using a log2-scale transformation to ensure standardisation. Any gene symbols detected using more than one probe were evaluated using their mean expression levels.

### Differential Expression Analysis and Enrichment Analysis of Oesophageal Cancer and Oesophageal Tissue

We merged 650 normal oesophageal tissue samples from the GTEx database and 77 oesophageal cancer samples from the TCGA-ESCC dataset using normalisation *via* R package “limma”. We then identified the differentially expressed Ras-related genes by comparing the tumour and precancerous tissue using a threshold false discovery rate (FDR) of <0.05, along with |log2 FC (fold-change) | > 2. GO and KEGG enrichment analyses were used to investigate the biological processes implicated by these differentially expressed genes using R package “clusterProfiler” were significance was set at *p* <0.05.

### Identification of Ras-Related Prognostic Genes

We used the data from previous studies to identify 180 critical Ras-related genes based on their KEGG database evaluations, of which 161 were included in the TCGA expression microarray data and thus used in our subsequent analysis. We then narrowed this to 11 genes which were shown to have some prognostic value based on a univariate Cox analysis with a threshold of *p* <0.05.

### Ras-Related Cluster and Clinical Correlation Analysis

We used the K-means algorithm to classify ESCC patients into different clusters based on 11 Ras-related prognostic genes expression (Hartigan and Wong, 1979), and the results showed that *K* = 3 was the best classification for all 77 TCGA patients in our cohort, producing Clusters 1 (*n* = 37), 2 (*n* = 21), and 3 (*n* = 19). The R package “ConsensusClusterPlus” was then applied to perform the above steps 1,000 times to guarantee the stability of the classification and we also verified the discriminatory power of these clusters using Kaplan-Meier survival analysis (Wilkerson and Hayes, 2010) and performed a correlation analysis with clinical features, which was visualized by R package “pheatmap”.

### Gene Set Variation Analysis and Functional Annotation

We then used Gene Set Variation Analysis (GSVA) enrichment analysis to investigate differences in these clusters across biological processes, using the “GSVA” R software package. GSVA is a non-parametric, non-supervised method commonly used to estimate changes in the activity of pathways and biological processes in samples from expression datasets. (Hänzelmann et al., 2013). The “c2.cp.kegg.v6.2.-symbols” gene sets were used to run these GSVA analyses, and were downloaded from the MSigDB database. Statistical significance was set at *p* <0.05.

### Investigation of Tumor Immune Microenvironment and Check Point Genes

We used CIBERSORT (http://cibersort.stanford.edu/) to evaluate the immune infiltration status of each of these three clusters, and the differences in their immune infiltration were then analysed using the Wilcoxon signed-rank test. At the same time, the ESTIMATE algorithm (R package “ESTIMATE”) was used to detect the activity of immune and stromal cells and evaluate tumour purity. (Yoshihara et al., 2013). We also performed a systematic search for immune checkpoint blockade gene expression profiles, such as PD-1, PD-L1, and CTLA-4 using the R packages “limma” and “ggpubr”.

### Establishment and Validation of Ras-Related Risk Assessment Model

To quantify the Ras modification pattern of individual tumours, we constructed a scoring system to assess the risk of ESCC patients, which we called the Ras Score. First, we performed multivariate Cox regression analysis with a threshold of *p* < 0.05 in 11 Ras-related prognostic genes, subsequently performed differential analysis of gene expression between the tumour and precancerous tissue with |log2 FC (fold-change) | > 2, in which three genes (EGFR, RAP1B, and PDGFRA) were identified for model development. The formula used to calculate the Ras score can be described as follows:

$$Ras\;Score={\hat{h}}_{0}\left(t\right)\sum_{i=1}^{k}\beta iSi$$

βi was the expression quantity of three genes (EGFR, RAP1B, and PDGFRA) and Si was coefficient of correlation of three genes. We selected the median value as the grouping criterion for the model to differentiate patients into high - and low-score groups.

We then validated this model by splitting the score distribution and survival status dot plots. At the same time, we used Kaplan-Meier survival analysis curves to evaluate differences in the survival statistics for the high and low Ras-score groups. This process was visualised using R packages “survival”, “glmnet”, “pbapply”, “survivalROC”, and “survminer”. The Ras-score was dichotomised by repeating the test on all possible cutpoints to find the surv-cutpoint function of the maximum rank statistic, and the patients were divided into high and low Ras-score groups according to the median to reduce the calculated batch effect.

### ROC Curves

The specificity and sensitivity of the Ras-score were assessed using a receiver operating characteristic (ROC) curve, and the area under the curve (AUC) was quantified using the pROC R package. The AUC for the ROC ranged from 0 to 1, with close to one indicating perfect predictive ability and 0.5 indicating no predictive ability, less than 0.5 indicating worse than random guesses.

### Chemotherapeutic Sensitivity Scoring

We then went on to explore the differences in chemotherapeutic sensitivity in the high- and low-score groups using “pRRophetic” in R to predict the half-maximal inhibitory concentration (IC50) of different chemotherapeutic drugs in each patient. This package predicts IC50 by creating statistical models based on drug sensitivity and RNA-Seq data based on the Genomics of Drug Sensitivity in Cancer (GDSC) (www.cancerrxgene.org/) database.

### Statistical Analysis

Analysis of the correlation coefficients between Ras and tumour microenvironment (TME)-infiltrating immune cell expression was performed using Spearman’s method and distance correlation analysis, respectively. One-way analysis of variance (ANOVA) and the Kruskal–Wallis test were used to compare the differences between three or more groups. (Hazra and Gogtay, 2016).

Univariate and multivariate Cox regression analyses were used to evaluate the correlations between various factors, including Ras-score and clinical characteristics. The log-rank test was used to compare survival differences between different groups and the waterfall function in the “maftools” package was used to visualized the mutation landscape in patients with high and low Ras-score subtypes in the TCGA-ESCC cohort. (Mayakonda et al., 2018). All Statistical *p* values were set at *p* < 0.05 and all data processing was performed using R version 4.0.2.

## Results

### Differential Expression of Genes and Functional Enrichment Analysis

**FIGURE 1:**
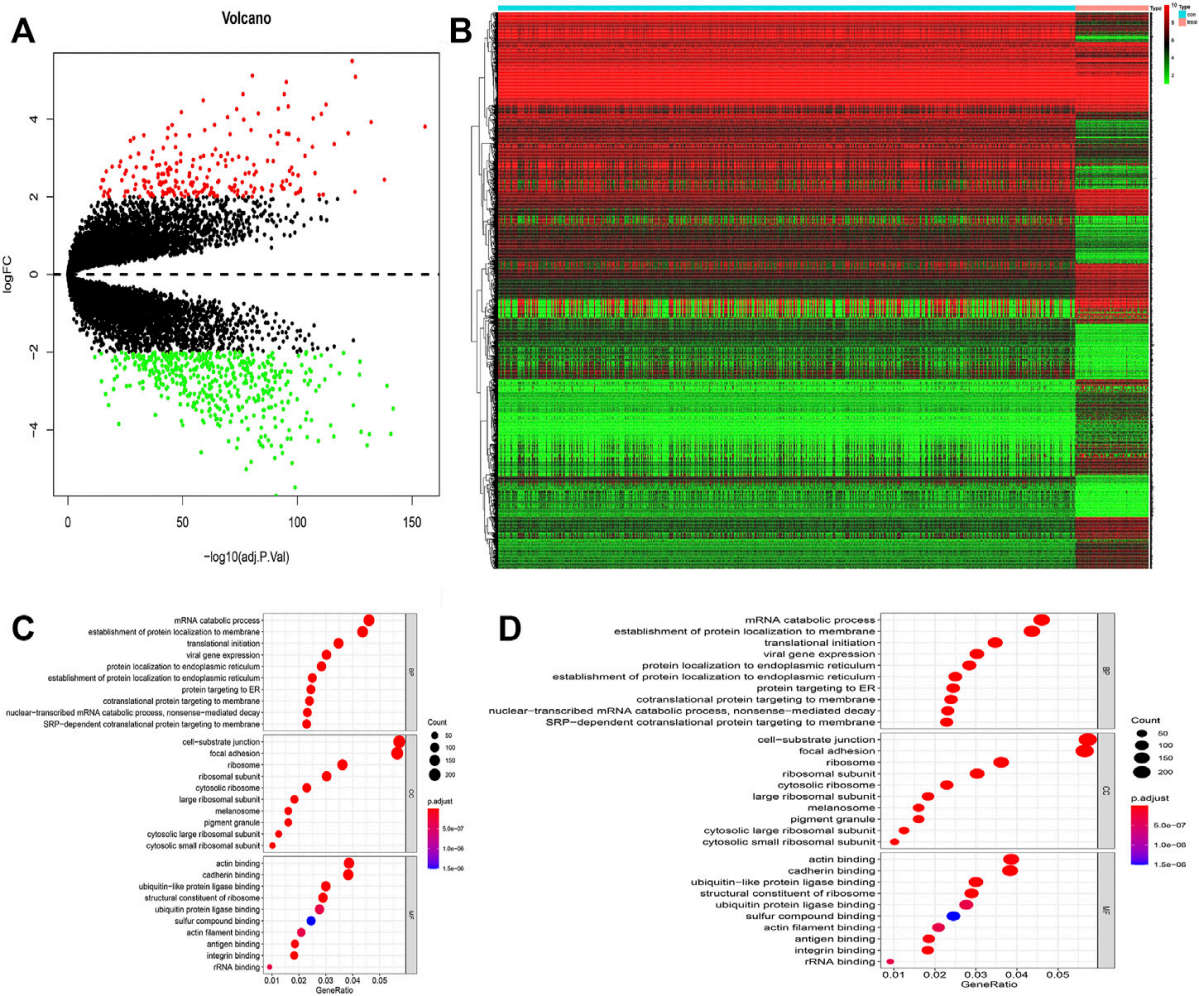
**(A)** Volcano map of different expressed genes between normal and ESCC samples. **(B)** Heatmap of different expressed genes between normal and ESCC samples. **(C–D)** Bubble plots of GO analyses **(C)** and KEGG analyses **(D)**. The larger bubble indicates the more obvious enrichment.

A total of 649 normal and 77 ESCC appropriate samples were retrieved from the TCGA and GTEx datasets. Our evaluation identified 4,568 significantly differentially expressed genes between the normal and ESCC samples which are represented in a heat- and volcano map (Figures 1A,B), respectively. These genes were then subjected to functional enrichment analysis designed to elucidate the biological functions and pathways of these differentially expressed genes. The GO results showed they were almost all enriched in cell-substrate junctions and focal adhesions (Figure 1C) and KEGG analysis showed that they were closely enriched in neurodegeneration-multiple diseases (Figure 1D).

### Genetic Expression of Ras-Related Genes in ESCC and Functional Enrichment Analysis

**FIGURE 2:**
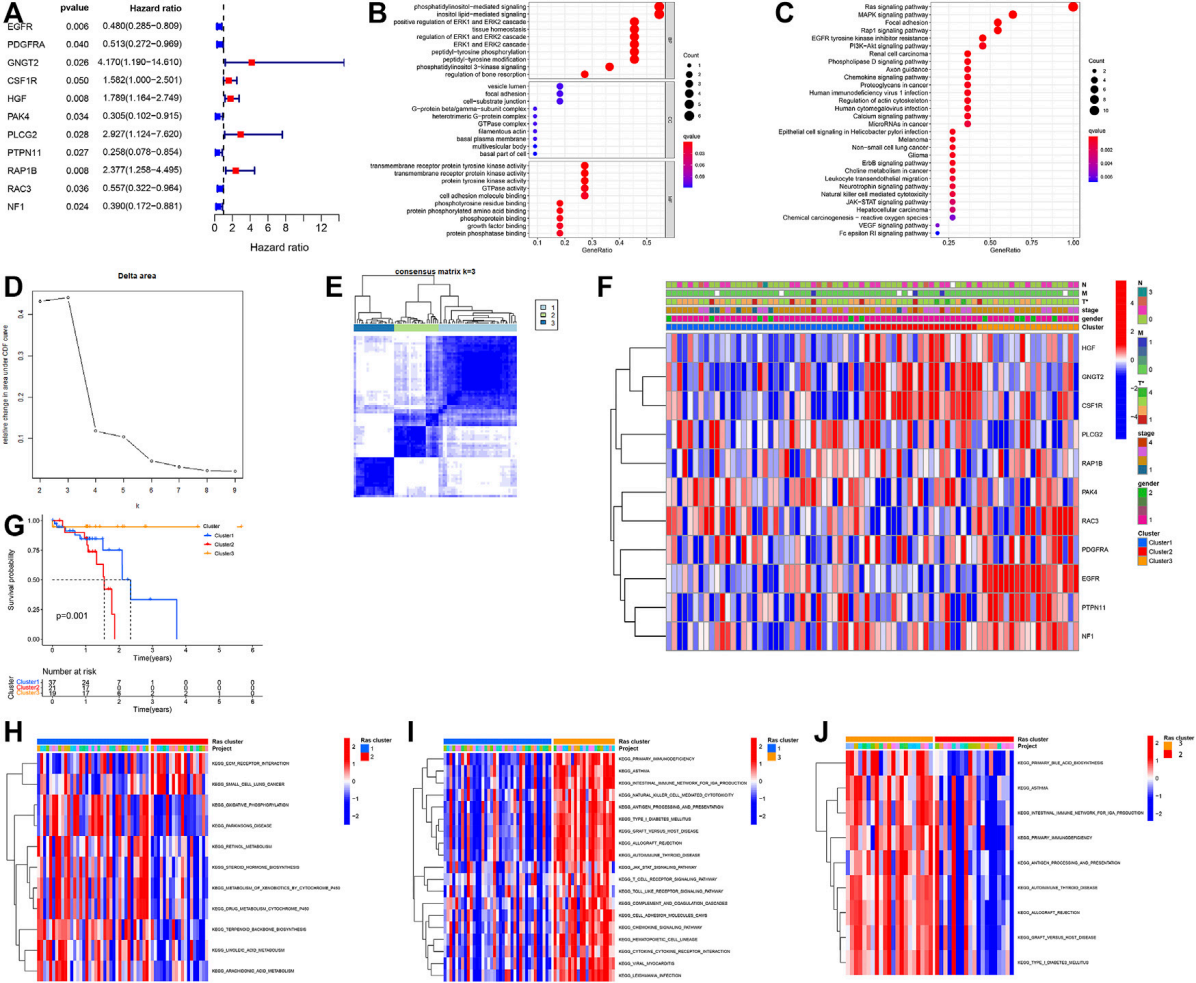
**(A)** Forest plot of hazard ratios exhibiting the prognostic worth of eleven Ras-related genes. **(B,C)** Bubble plots of GO analyses **(B)** and KEGG analyses **(C)**. **(D,E)** Unsupervised clustering of 11 Ras-related prognostic genes in the TCGA-ESCC cohort. **(F)** Heatmap of Ras-related genes. Blue represents down-regulation and red represents up-regulation of genes. **(G)** Kaplan–Meier survival curve. **(H–J)** GSVA enrichment analysis showing the activation states of biological pathways in distinct Ras modification patterns. The heatmap was used to visualize these biological processes, and red represented activated pathways and blue represented inhibited pathways. **(H)** Ras-cluster1 vs Ras-cluster 3; **(I)** Ras-cluster1 vs Ras-cluster 2; **(J)** Ras-cluster3 vs Ras-cluster two.

Our study cohort was made up of a single TCGA dataset (TCGA-ESCC) which included both the gene expression profile of these samples but also the relevant OS and clinical data needed for this kind of evaluation. We used this data in our univariate Cox regression evaluation of 161 Ras-related genes and identified 11 Ras-related genes with probable prognostic value when screened using a p of <0.05 (Figure 2A). We then performed functional enrichment analysis to elucidate the biological functions and pathways of 11 prognostic Ras-related genes. GO results showed that ERK1 and ERK2 cascade, cell adhesion molecule binding, and transmembrane receptor protein tyrosine kinase were significantly enriched (Figure 2B). Ras signaling pathway, MAPK signaling pathways, Rap1 signaling pathways, and EGFR tyrosine kinase inhibitor resistance were significant KEGG enrichment items (Figure 2C).

### Ras Modification Patterns Are Predicted by 11 Ras-Related Prognostic Genes

We used the Unsupervised clustering analysis to categorise patients into different Ras-related gene clusters within the TCGA-ESCC cohort based on their expression of each of the 11 Ras-related prognostic genes. The change curve of the consensus cluster cumulative distribution function from *k* = 2 to nine showed that the area under the curve was the largest when *k* = 3 (Figure 2D), so we produced three different modification patterns, including Ras-cluster 1 (*n* = 37), Ras-cluster 2 (*n* = 21), and Ras-cluster 3 (*n* = 19) (Figure 2E). Prognostic analysis of these three clusters revealed an obviously significant survival advantage for Ras-cluster 3, and the worst survival for Ras-cluster 2 (Figure 2G). There was also a significant difference in the Ras gene expression profile among the three clusters (Figure 2F). Ras-cluster one was characterised by the increased expression levels of PLCG3, RAP1B, and PAK4, and presented with variable decreases in other Ras-related prognostic genes; Ras-cluster two showed high expression levels of HGF, GNGT2, and CSF1R and Ras-cluster three exhibited a significant increase in the expression of RAC3, PDGFRA, EGFR, PTPN11, and NF1.

### Gene Set Variation Analysis and Differences in Immune Characteristics Between Clusters

Given these outcomes we then used GSVA to explore differences in the biological behaviours of these three clusters. These results showed that all three clusters were all using dramatically different immune signalling pathways. Ras-cluster two presented with in enrichment in the pathways associated with full immune activation, including the activation of the chemokine and T cell receptor signalling pathways, cytokine-cytokine receptor interaction, and Toll-like receptor signalling pathways (Figures 2H–J).

**FIGURE 3:**
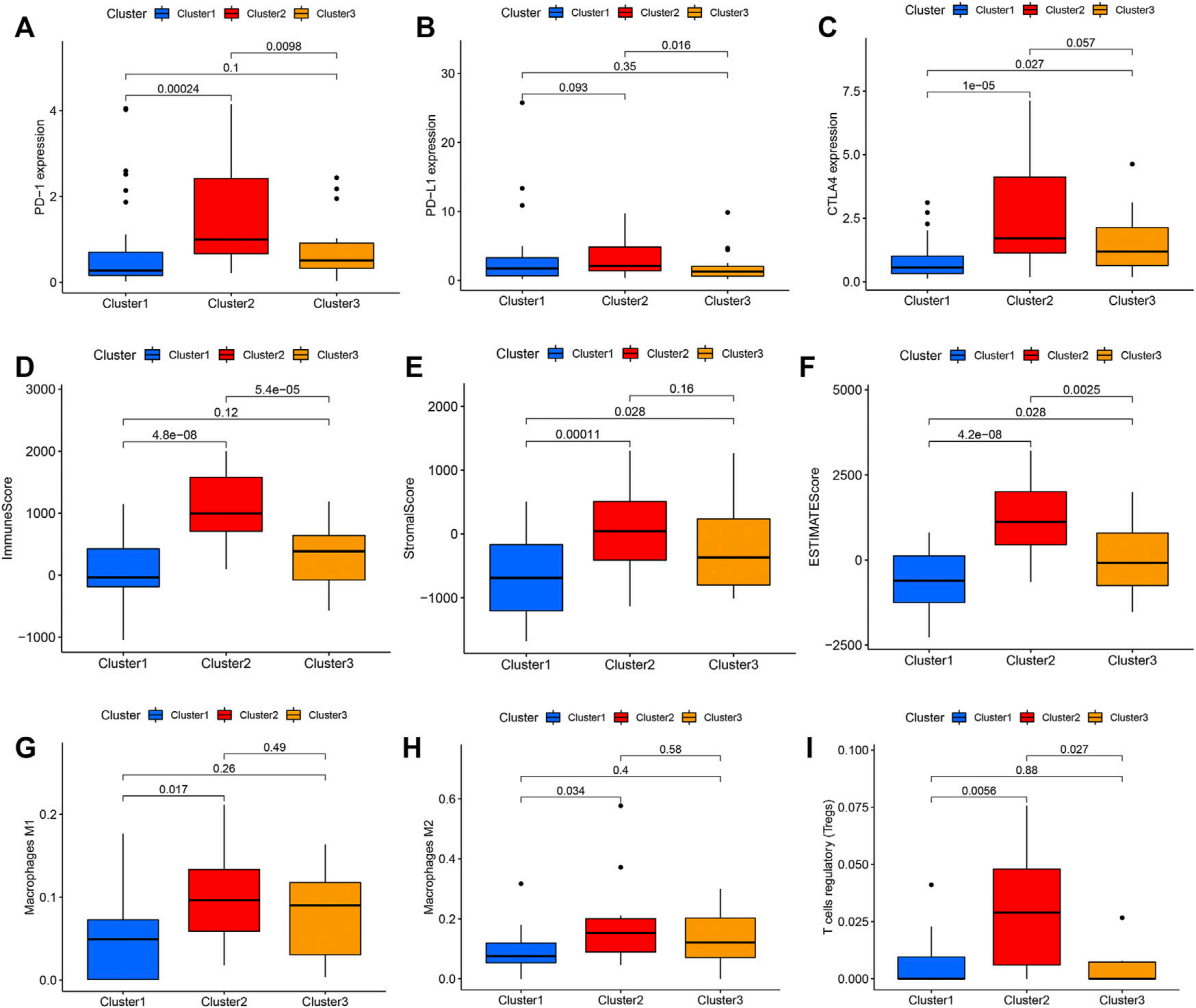
Differential expression of immune checkpoints in Ras-cluster1 vs Ras-cluster 2 vs Ras-cluster three patients. The upper and lower ends of the boxes represented interquartile range of values. The lines in the boxes represented median value, and black dots showed outliers. The asterisks represented the statistical *p* value (ns *P* > 0.05; *p < 0.05; **p < 0.01): **(A)** PD-1, **(B)** PD-L1, **(C)** CTLA4, **(D)** Immune score, **(E)** Stromal score, **(F)** ESTIMATE score, **(G)** Macrophages M1, **(H)** Macrophages M2, **(I)** T cells regulatory.

Thus, we went on to compare the expression of the immune checkpoint genes in these three clusters and found significant differences in PD-1, PD-L1, and CTLA4 expression in each. The expression level of PD-1, PD-L1, and CTLA4 was significantly higher in the Ras-cluster2 (Figures 3A–C) as were the number of immune and stromal cells when comparing Ras-cluster two and Ras-clusters one and 3 (Figures 3D–F). The purity of the cancer cells in Ras-cluster2 was low and immune cell infiltrations with M1 and M2 macrophages and T cells was significantly higher in Ras-cluster 2. These data were all consistent with the immune checkpoint expression and immune scores for these pathological groups (Figures 3G–I).

### Reliability of the Novel Three Ras-Associated Gene Signature

**FIGURE 4:**
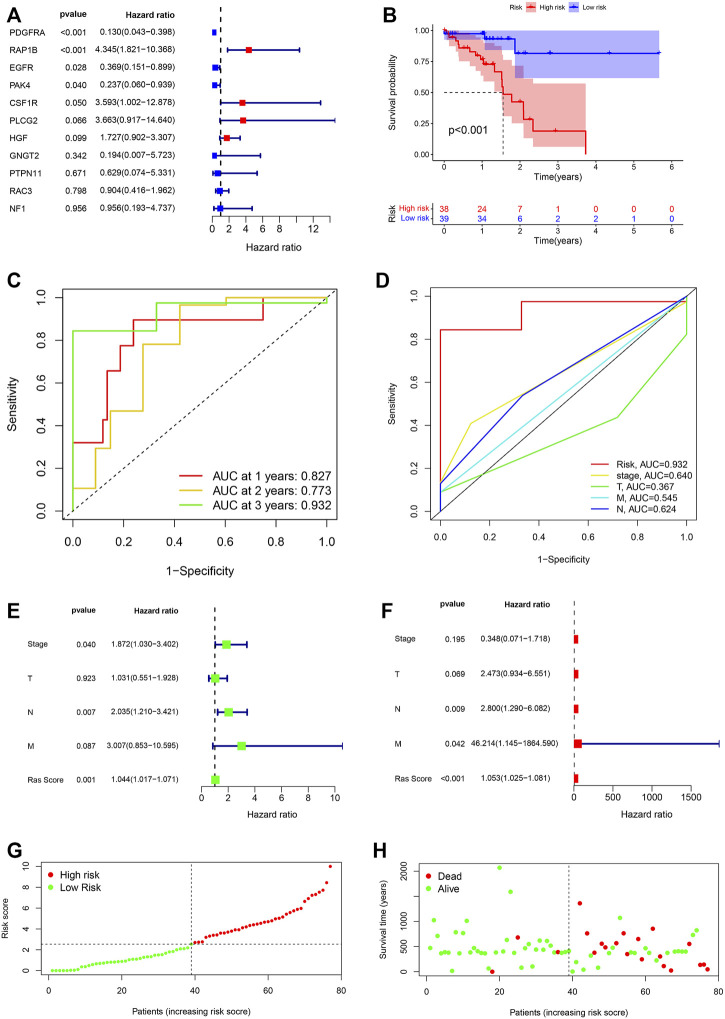
**(A)** The prognostic Ras-related genes extracted by multivariate Cox regression analysis. **(B)** Kaplan-Meier analysis on high-Ras score and low-Ras score patients. **(C)** Time-dependent ROC curve analyses of Ras score. **(D)** ROC curve analyses of TNM status, stage and Ras score. **(E,F)** Uni- and multi-Cox analyses of clinical factors and risk score with OS. **(G)** Ras score distribution of high-Ras and low-Ras score patients. **(H)** Survival status of high- and low-Ras score patients.

We performed multivariate Cox regression analysis and differential analysis of gene expression between the tumour and precancerous tissue on 11 prognostic Ras-related genes, and we got three differently expressed prognostic Ras-related genes (EGFR, RAP1B and PDGFRA) (Figure 4A). We then went on to build a Ras-related risk assessment model for predicting the OS of ESCC patients. We did this by constructing a set of scoring systems based on the three most relevant Ras-related prognostic genes (EGFR, RAP1B and PDGFRA) and use these to quantify the Ras modification pattern of individual patients giving them a Ras-score. The formula for calculating the Ras-score for each ESCC patient is as follows: Ras − score = −0.027390631097796 ∗ EGFR − 0.250341831449203 ∗PDGFRA + 0.0669651154761456∗RAP1B.

We then went on to evaluate the value of these Ras-scores in predicting specific patient’ outcomes, and these evaluations identified that patients with a low Ras-score experienced significantly better survival rates (Figure 4B). We then used ROC analysis to determine the credibility of our model for predicting prognosis. Our ROC AUC values for the one-, two-, and 3-year survival of our TCGA-ESCC cohort were determined to be 0.827, 0.773, and 0.932, respectively, (Figure 4C). This same analysis was then conducted within the cohort, and the AUC values for stage, T, M, and N in 3-year survival were 0.640, 0.367, 0.545, and 0.624, respectively, (Figure 4D).

We then tested whether the Ras-score could serve as an independent prognostic biomarker for ESCC. We demonstrated that stage [*p* = 0.04, *HR* = 1.872, 95%CI (1.030–3.402)], T stage [*p* = 0.923, *HR* = 1.03 1, 95% CI (0.551–1.804)], N stage [*p* = 0.007, *HR* = 2.035, 95% CI (1.210–3.421)], M stage [*p* = 0.087, *HR* = 3.007, 95%CI (0.853–10.595)], and Ras-score [*p* = 0.001, *HR* = 1.044, 95% CI (1.017–1.071)] showed significant differences in univariate Cox regression analysis (Figure 4E), whereas only N stage [*p* = 0.009, *HR* = 2.800, 95% CI (1.290–6.082)], M stage [*p* = 0.042, *HR* = 46.214, 95% CI (1.145–1864.590)], Ras-score (*p* <0.001, *HR* = 1.053, 95% CI (1.025–1.081)] presented as independent prognostic predictors in multivariate Cox regression analysis (Figure 4F). Multivariate Cox regression model analysis confirmed that the Ras-score could act as an independent and robust prognostic biomarker for evaluating patient survival in ESCC. We classified the ESCC patients into low- and high-Ras score groups based on the median Ras score (Figure 4G) and the predictive performance of our Ras score model for predicting patient OS is shown in Figure 4H.

### Differences in Mutated and Key Gene Expression Between High and Low Ras-Score Groups

**FIGURE 5:**
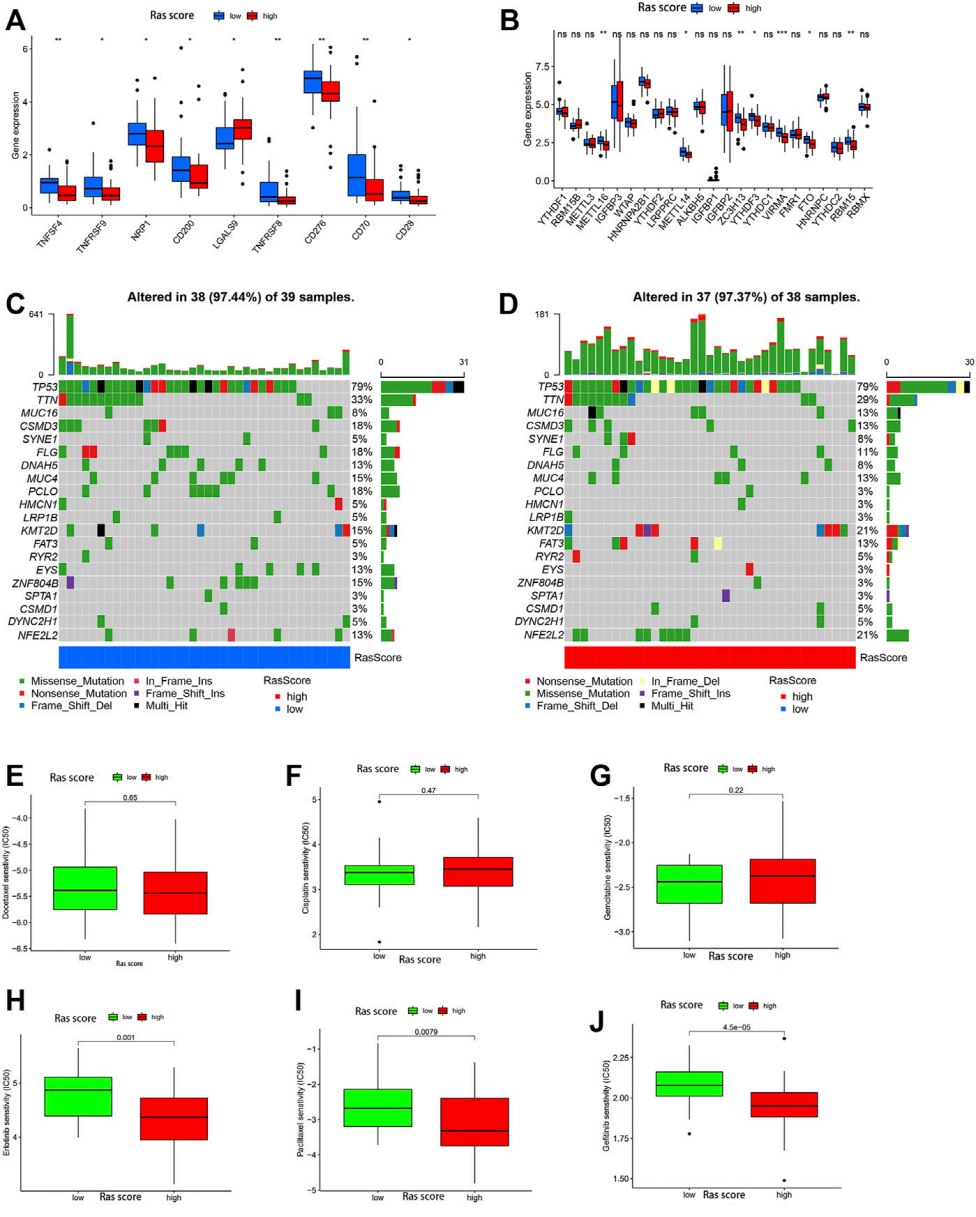
**(A,B)** The expression of 30 immune checkpoints genes in high-Ras and low-Ras score patients. The upper and lower ends of the boxes represented interquartile range of values. The lines in the boxes represented median value, and black dots showed outliers. The asterisks represented the statistical *p* value (ns *P* > 0.05; *p < 0.05; **p < 0.01). The one-way ANOVA test was used to test the statistical differences among high-Ras score and low-Ras score patients. **(C,D)** The waterfall plot of tumor somatic mutation established by those with low Ras-score **(C)** and high Ras-score **(D)**. **(E–J)** The sensitivity to drugs in high-Ras and low-Ras score patients: **(E)** Docetaxel, **(F)** Cisplatin, **(G)** Gemcitabine, **(H)** Erlotinib, **(I)** Paclitaxel, **(G)** Gefitinib.

Given these outcomes, we further explored the relationship between the important immune-gene predictors and the two Ras-score groups using differentiation analysis. In this evaluation, we compared the expression levels of several critical immune checkpoint genes, including TNFSF4, TNFRSF8, TNFRSF9, NRP1, CD28, CD70, CD200, CD276, METTL16, METTL14, ZC3H13, YTHDF3, VIRMA, FTO, and RBM15 in each of the Ras-score groups (Figures 5A,B) and found that each of these genes were significantly upregulated in the low-Ras-score group when compared to the high-Ras score group, while LGALS9 was enriched in the high-Ras-score group.

We also used the gene mutation data from the 77 ESCC patients in our dataset to analyse the difference in gene mutations between these two groups, and the genes with the top 20 mutation rates are shown in Figures 5C,D. The results showed that CSMD3, FLG, DNAH5, MUC4, PLCO, EYS, and ZNF804B experienced significantly higher mutation rates in the low-Ras-score group, whereas MUC16, KMT2D, FAT3, and NFE2L2 were highly mutated in the high-Ras-score group.

### Correlation Analysis Between Ras-Score and Chemosensitivity

We then evaluated the utility of this score in predicting drug sensitivity in ESCC patients in the high and low-Ras groups. These results showed that there was no significant difference in the sensitivity of these samples to traditional chemotherapeutic drugs such as cisplatin, gemcitabine and docetaxel between the high and low Ras-score groups (Figures 5E–G), while paclitaxel had better sensitivity in the high Ras-score group. It is worth noting that targeted drugs such as erlotinib and gefitinib also had better sensitivity in the high Ras-score group (*p* <0.05) (Figures 5H–J).

## Discussion

ESCC is a progressive disease with a poor prognosis. At present, clinicians primarily use the TNM staging system to evaluate the prognosis of patients with cancer. However, most studies have revealed heterogeneity in the prognosis of tumours at the same stage. Accumulating evidence suggests that the Ras mutation rate can regulate the process of tumour development and progression, and is also involved in regulating the immune response to these tumours. (Masliah-Planchon et al., 2016; Ryan and Corcoran, 2018; Zhang et al., 2018; Chen et al., 2019; Prior et al., 2020). However, systematic analysis of RAS in ESCC is still rare, and the underlying mechanism remains unclear. (Feng et al., 2018; Li et al., 2019; Feng et al., 2020).

Our initial investigation identified 11 special survival-related Ras-associated genes which could be used to classify three distinct Ras modification patterns. There was a significant difference in the Ras gene expression profile between each of these three patterns and our evaluations revealed that Ras-cluster three had a particularly significant survival advantage over both Ras-clusters two and 1. Ras-cluster three exhibited significant increases in the expression of RAC3, PDGFRA, EGFR, PTPN11, and NF1. This correlates with the current therapeutic focus on EGFR which includes clinical trials for advanced EC with high HER-2 expression, showing the therapeutic potential of EGFR targets in oesophageal cancer. (Bang et al., 2010; Yan-Ming Yang et al., 2020; Moehler et al., 2020). PDGFRA has demonstrated its potential as a therapeutic target for gastrointestinal stromal tumours (Theiss and Contreras, 2019) and these studies are consistent with the results of our study which suggest that the increased expression of these genes improves the survival potential of patients in Ras-cluster 3.

PD-1, PD-L1, and CTLA4 expression levels were also all increased in Ras-cluster three when compared with the others, while Ras-cluster two had a much higher rate of immune and stromal cell infiltration than either Ras-cluster one or Ras-cluster 3. This included a significant increase in the number of M1 and M2 macrophages and T cells in Ras-cluster2, which was consistent with the immune target expression and immune scores for these patients. Previous studies have shown that tumours with an immunoinflammatory phenotype also exhibit large numbers of stromal and immune cells despite the fact that stromal activation in the TME is thought to be inhibited by increased numbers of T-cells. (Chen and Mellman, 2017). Ras-cluster two was characterised by an immunoinflammatory type, specifically characterised by adaptive immune cell infiltration and immune activation, resulting in likely immune depletion in these patients, which might explain its poor survival potential.

We used the TCGA-ESCC and GTEx-normal sets to identify differentially expressed genes and then specific prognostic Ras-related genes using multiple Cox regression analysis and then used these to build a predictive model for ESCC comprising just three differentially expressed Ras-associated genes.

Kaplan-Meier curve analysis showed that cases in the high Ras-score group were associated with reduced OS when compared with patients in the low Ras-score group. Our ROC values illustrate the solid performance of this prognostic model, with the AUC of these curves for 3-years OS in the TCGA cohort being recorded at 0.932. In addition, we used these ROC curves to compare the predictive efficacy of our proposed model with other clinical features and revealed that our model was more predictive than other prognostic factors for ESCC, such as gender, stage, T stage, N stage and M stage.

It has been shown that KMT2D mutations are associated with increased tumour size and unfavorable prognosis in patients with EC. (Zheng et al., 2021). A phase 3 trial of mantle cell lymphoma showed that mutations in KMT2D mutation were associated with an increased risk of death in these patients. (Ferrero et al., 2020). FAT3 mutations are related to poor prognosis in oesophageal cancer, (Guo et al., 2021), while NFE2L2 mutations were significantly associated with a worse prognosis in ESCC. (Cui et al., 2020). In patients with oesophageal cancer, metabolic reprogramming of the glutathione metabolism, as well as detoxification of ROS by activation of NFE2L2, enhances cancer progression, leading to poor clinical outcomes. (Kitano et al., 2018). These results explain why the prognosis of patients was worse in the high Ras-score group with higher rates of certain gene mutations, such as KMT2D, FAT3, and NFE2L2, further confirming the accuracy and effectiveness of the Ras-score model in predicting patient prognosis. Our results show that the Ras score is a good prognostic index for ESCC when compared to traditional staging. The results that paclitaxel, erlotinib and gefitinib had better sensitivity in the high Ras-score group also show the capacity of Ras score to predict these drugs treatments value in ESCC patients. However, this study might still have its limitations, especially given that the number of training set samples was relatively small (78 samples) and a validation set was not available.

In summary, we identified differentially expressed Ras-related genes that may be involved in ESCC pathogenesis. These genes are of significant value in predicting OS in ESCC patients and may provide new options for individualized therapy. Further studies are necessary to verify the results of our evaluations and future work should include both *in vitro* and *in vivo* validations.

## Data Availability

The original contributions presented in the study are included in the article/Supplementary Material, further inquiries can be directed to the corresponding authors.
